# Neonicotinoids act like endocrine disrupting chemicals in newly-emerged bees and winter bees

**DOI:** 10.1038/s41598-017-10489-6

**Published:** 2017-09-08

**Authors:** Danica Baines, Emily Wilton, Abbe Pawluk, Michael de Gorter, Nora Chomistek

**Affiliations:** Agriculture and Agri-Food Canada, Lethbridge Research and Development Centre, 5403 1 Avenue South, Lethbridge, Alberta T1J 4B1 Canada

## Abstract

Accumulating evidence suggests that neonicotinoids may have long-term adverse effects on bee health, yet our understanding of how this could occur is incomplete. Pesticides can act as endocrine disrupting chemicals (EDCs) in animals providing characteristic multiphasic dose-response curves and non-lethal endpoints in toxicity studies. However, it is not known if neonicotinoids act as EDCs in bees. To address this issue, we performed oral acute and chronic toxicity studies including concentrations recorded in nectar and pollen, applying acetamiprid, clothianidin, imidacloprid, and thiamethoxam to bumble bees, honey bees and leafcutter bees, the three most common bee species managed for pollination. In acute toxicity studies, late-onset symptoms, such as ataxia, were recorded as non-lethal endpoints for all three bee species. Clothianidin and thiamethoxam produced biphasic dose-response curves for all three bee species. Clothianidin and thiamethoxam were extremely toxic to winter worker honey bees prior to brood production in spring, making this the most sensitive bee stage identified to date. Chronic exposure to field-realistic levels of neonicotinoids reduced bee survival and caused significant late-onset symptoms for all three bee species. Given these findings, neonicotinoid risk should be reevaluated to address the EDC-like behavior and the sensitivity of winter worker honey bees.

## Introduction

Bees maintain biodiversity and agricultural productivity by pollinating a wide range of flowering crops and wild plants. Since 2008, higher honey bee colony losses have been recorded internationally with the largest losses attributed to winter kill^1–3^. Several factors contribute to honey bee colony losses including stress, queen failure, treatment with acaricides for Varroa mite management, and access by foraging honey bees to nutritional crops^3^, but changes in the patterns of insecticide usage have also coincided with these losses suggesting but not proving causation^1–7^. For example, neonicotinoid applications have been linked with localized honey bee colony losses and poor overwintering bee survival, particularly with dust created from sowing neonicotinoid-coated seeds^8–11^. Among wild bees, neonicotinoid applications have also been linked with changes in bee populations including wild bee density, nesting, colony growth and reproduction^12–15^. If neonicotinoids are contributing to these global changes in bee populations, the answer may lie in the insecticide usage patterns transitioning from the first generation neonicotinoid, imidacloprid, to the second generation neonicotinoids, clothianidin and thiamethoxam. Neonicotinoids target nicotinic acetylcholine receptors (nAChR) in bees, however thiamethoxam also interacts with muscarinic acetylcholine receptors (mAChR)^16–18^. Acute early-onset symptoms resulting from neonicotinoid interactions with target receptors have been reported for worker honey bees, but only at unrealistic field concentrations^19–22^. For example, clothianidin acts as a super-agonist opening nAChR channels and a common neurological symptom is hyperactivity in foraging worker honey bees. Very little attention has been paid to delayed-onset symptoms resulting from acute or chronic exposure to field-realistic concentrations of neonicotinoids.

Several features of oral acute and chronic toxicity studies applying neonicotinoids to three managed bee species affected the information generated including the length of the assessment period, the dose range applied and the dosing procedure^6, 19–22^. The first lab-based acute toxicity studies investigated contact toxicity using formulated neonicotinoid products where a dose was applied dorsally on the thorax of mixed nurse- (1 to 22 days old) and forager-aged (23–42 days old) worker honey bees^20^. These studies established that there were no significant differences between the LD_50_ values among commercial formulations for contact toxicity, but this was likely a consequence of the formulation which facilitates cuticular transmission rather than innate chemical properties of the pure neonicotinoids. In subsequent lab-based oral acute toxicity studies with mixed-age worker honey bees, formulated imidacloprid was about ten times less toxic than clothianidin or thiamethoxam^19, 21, 22^. Based upon the LD_50_ values generated in the acute toxicity studies, it was predicted that imidacloprid should have low toxicity for worker honey bees in semi-field or field conditions. However, in practice, this was not the case^8, 9^ and undermined the value of the results for predicting adverse effects in the environment. This same discrepancy was recorded with worker bumble bees^23, 24^.

Worldwide, pollen and nectar vary with respect to the types and concentrations of neonicotinoids recovered. For example, imidacloprid was detected between 0 and 5 µg/kg in plant samples, 912 ng/g in pollen samples or 2 ng/g in honey samples, all of which are well below LD_50_ values for imidacloprid for worker honey bees suggesting but not proof of a low risk to populations^25–27^. Juxtaposed to these values, up to 27 µg/kg imidacloprid or 3.24 ng/honey bee sample has been detected in bees displaying neurological symptoms such as trembling or clustering outside the colony^28^. At field-realistic concentrations, the most common early-onset symptoms in pest insects and worker honey bees are behavioral changes including reduced responses to sex pheromones^29^, impaired associative learning^30^, impaired short-term memory^31^, and impaired movement^32^. These behavioral changes were also observed in oral chronic toxicity studies applying field-realistic concentrations of thiamethoxam to bumble bee colonies^33^. There is no evidence that lab-based acute and chronic toxicity studies were repeated with managed bees to determine whether these behavioral changes were permanent and always linked with neonicotinoid exposure thereby, defining them as non-lethal endpoints^34^ for toxicity assessments.

EDCs are hormone-like chemicals that interfere with or prevent natural hormone activities in the body^35^ and include persistent pesticides such as neonicotinoids^36^, but the term is mainly applied to chemicals, such as polychlorinated biphenyls^37^, affecting vertebrate and environmental health. The most common quantitative feature of EDCs is the multiphasic dose-response curve which can result in a stimulatory response at environmental concentrations that are associated with 30–60% mortality relative to untreated animals. The concentrations necessary to document the multiphasic nature of neonicotinoids has not been applied in any bee toxicity studies. EDCs elicit a number of irreparable neurological symptoms that are applied to mortality or survival estimates in toxicity studies. Oral chronic toxicity studies with neonicotinoids applied to foraging worker honey bees, and other invertebrates demonstrate neurological symptoms including abnormal behavior^29–33^, reproductive^38^, development^39^ and immune function^40^, but it is not clear whether these changes are permanent and should be included in toxicity studies as non-lethal endpoints. An EDC target that may explain winter kill in bees^1–3^, hormone status^41–45^, has not been investigated with bees despite evidence of a role with other pesticides^35^. For example, neurohormones and their receptors are downregulated in winter worker honey bees making them less susceptible to insecticide exposure in the fall^46^. No studies have examined the outcome of winter honey bee susceptibility to neonicotinoids in spring, when the hormone systems are upregulated.

To determine if neonicotinoids act as EDCs in bees, we expanded the doses applied in acute and chronic toxicity studies to include concentrations equivalent to natural agonists of hormone receptors in insects, such as juvenile hormone (picomole), and lengthened the assessment period. This ensured that time-based neurological symptoms are recorded and added, where appropriate, as an endpoint in toxicity assessments. To address the possible role of species on bee susceptibility, we examined the oral acute and chronic toxicity of four neonicotinoids in worker bumble bees (*Bombus impatiens*), worker honey bees (*Apis mellifera*) and leafcutter bees (*Megachile rotundata*). To address the role of hormone status on bee susceptibility, we compared oral acute and chronic toxicity studies using four neonicotinoids in newly-emerged honey bees and newly-emerged leafcutter bees with previous studies using mature worker honey bees and leafcutter bees. To further validate the significant role of hormone status to susceptibility, we performed acute toxicity studies with the four neonicotinoids applied to winter worker honey bees in spring.

## Results

### Experiment 1: Acute exposure in bumble bees

**Figure 1:**
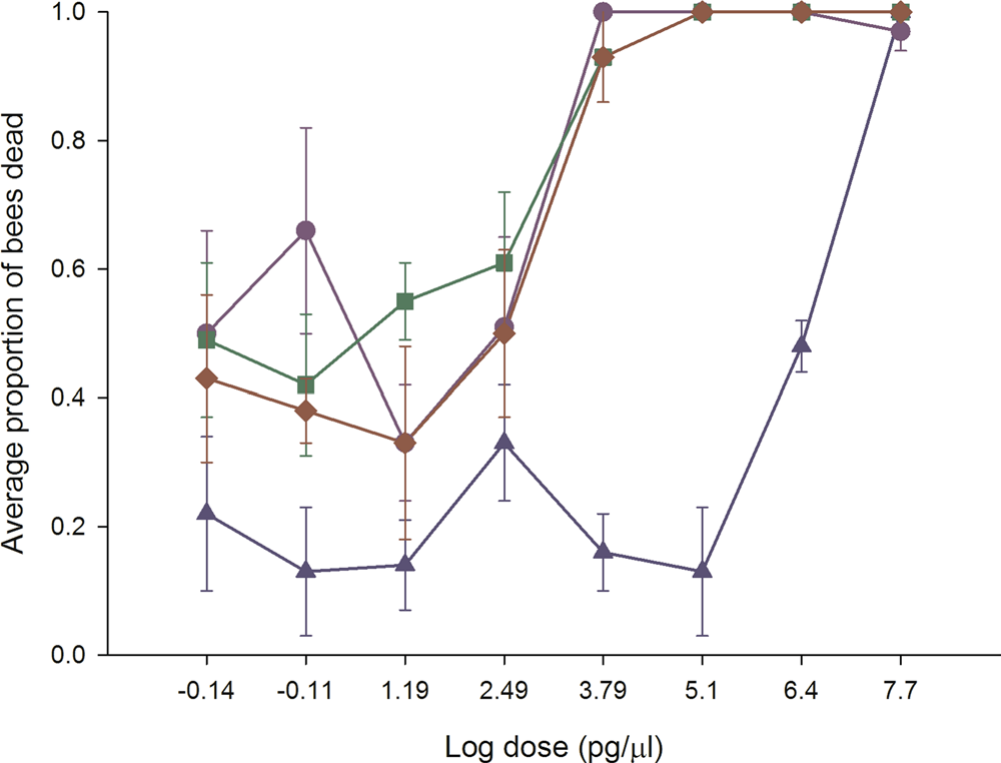
Clothianidin, imidacloprid and thiamethoxam elicit biphasic dose-response curves in bumble bees. Bumble bees were provided untreated honey-water or honey- water containing 50 µg/µl, 2.5 µg/µl, 125 ng/µl, 6.25 ng/µl, 312.5 pg/µl, 15.6 pg/µl, 0.78 pg/µl and 0.039 pg/µl acetamiprid, imidacloprid, thiamethoxam and clothianidin for 24 hours in cage trials. The cages were then provided honey water ab libitum for 14 days. As a conservative estimate, we used the highest consumption rate or 120 µl for all LD_50_ calculations. Cages were assessed daily for the number of bees dead in a cage, the number of bees exhibiting symptoms, the type of symptoms, and the duration or outcome of symptoms. The average values and standard errors are provided for each concentration applied. The data were collected from 6 independent experiments and expressed as proportion of bees dead. The four neonicotinoids are identified as follows: acetamiprid (blue triangle), clothianidin (red diamond), imidacloprid (green square) and thiamethoxam (purple circle).

Oral acute toxicity studies with acetamiprid (χ^2^ = 67.16, df = 8, *P* < 0.01) resulted in a monotonic dose-response curve with bumble bees (Fig. 1). This neonicotinoid was non-toxic at field realistic concentrations or the lowest two doses applied (0.039–0.78 µg/µl). In contrast, clothianidin (χ^2^ = 319.6, df = 8, *P* < 0.01), imidacloprid (χ^2^ = 119.12, df = 8, *P* < 0.01) and thiamethoxam (χ^2^ = 113.19, df = 8, n = 48, *P* < 0.01) exposure resulted in biphasic dose-response curves illustrating an EDC-like behavior. The curves are U-shaped and were most pronounced with thiamethoxam, with an average mortality of 58% between 6 pM to 133 pM (0.039–0.78 pg/µl), 33% to 51% between 3 nM to53 nM (15.6–312.5 pg/µl) and 100% at 1 µM (1.1 pg/µl). There was a significant colony effect (*P* ≤ 0.05) indicating that the amount of bee losses was related to the colony from which the bees were collected. Low levels of mortality were recorded within the Organisation for Economic Co-operation and Development (OECD) guidelines of 96 hr for acute toxicity bioassays, but mortality peaked at 7 days post-challenge compared with untreated bees. Acute early-onset neurological symptoms (minutes to 24 hours) resulting from high exposure doses (50 µg/µ/ to 6.25 ng/µl), were specific to each neonicotinoid: clothianidin and thiamethoxam caused hyperactivity; clothianidin and imidacloprid caused trembling; clothianidin, imidacloprid and thiamethoxam caused excessive grooming; clothianidin and imidacloprid caused uncontrolled proboscis extension; acetamiprid and imidacloprid caused slow to no movements. Delayed-onset symptoms (5 to 7 days after exposure) recorded for moderate (ng/µl) to low (pg/µl) exposure doses were also specific to each neonicotinoid: clothianidin and thiamethoxam caused intermittent hyperactivity; acetamiprid, imidacloprid and thiamethoxam caused abnormal stance and slow movements. In all cases, the delayed-onset symptoms progressed to abnormal placement of the legs/wings and an ataxia that made the bee superficially look like a spider. Although food was available, individuals displaying these symptoms did not recover normal daily activities or consume food, but remained alive for the 14 day assessment period. This type of non-lethal endpoint is not uncommon in toxicology studies^34^, and is a feature of EDC behavior^35, 37^. If we score these neurologically-impaired bees as dead, the entire dose-response curve shifts to the left by a minimum of two positions. These results recommend the extension of the OECD guideline assessment period to 7 days to capture adverse effects on bumble bees.

The threshold dose or NOAEL (no observed adverse effect level) was derived from the dose-response curves. The NOAEL was 37.5 ng/bee for acetamiprid, 1.9 ng/bee for clothianidin, 0.93 ng/bee for imidacloprid and 0.93 ng/bee for thiamethoxam. The LD_50_ value was 300 µg/bee for acetamiprid and is equivalent to the second highest dose applied. A single LD_50_ value could not be generated for clothianidin, imidacloprid and thiamethoxam as there were two peaks associated with bee mortality. Therefore, for these chemicals two LD_50_ values could be calculated: 94.5 pg/bee and 1.87 ng/bee for clothianidin; 0.23 pg/bee and 4.69 pg/bee for imidacloprid; 22.6 pg/bee and 98.2 pg/bee for thiamethoxam. The relative toxicity of the neonicotinoids was: imidacloprid≫ thiamethoxam > clothianidin≫ acetamiprid. Assuming a daily consumption rate of 120 µl of contaminated food, clothianidin, imidacloprid and thiamethoxam occur in sufficient quantities in natural bee food to have adverse effects on bumble bees.

### Experiment 2: Chronic exposure in bumble bees

**Figure 2:**
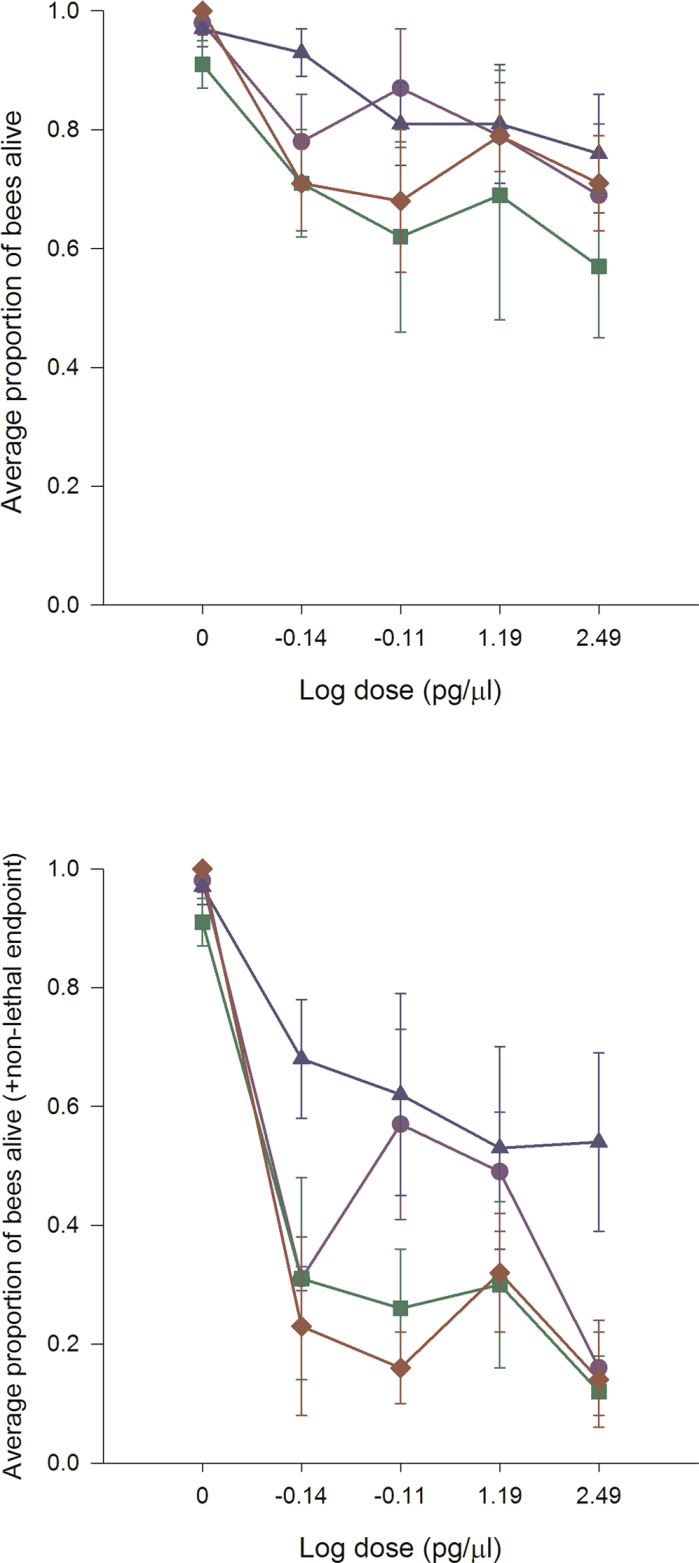
Clothianidin, imidacloprid and thiamethoxam decrease bumble bee survival at field relevant levels. Bumble bees were provided untreated honey-water or honey- water containing 312.5 pg/µl, 15.6 pg/µl, 0.78 pg/µl and 0.039 pg/µl acetamiprid, imidacloprid, thiamethoxam and clothianidin for 14 days in cage trials. As a conservative estimate, all calculations were done using the highest consumption rate or 120 µl. Cages were assessed daily for the number of bees alive in a cage, the number of bees exhibiting symptoms, the type of symptoms, and the duration or outcome of the symptoms. The average values and standard errors are provided for each concentration applied. The data were collected from 6 independent experiments and expressed as proportion of bees alive. The four neonicotinoids are highlighted as follows: acetamiprid (blue triangle), clothianidin (red diamond), imidacloprid (green square) and thiamethoxam (purple circle). The top graph indicates the bee survival without non-lethal endpoints added while the bottom graph indicates the bee survival with non-lethal endpoints added.

In oral chronic toxicity studies, applying the four neonicotinoids to bumble bees resulted in a dose-dependent decline in survival for clothianidin (*P* = 0.001), imidacloprid (*P* = 0.003) and thiamethoxam (*P* = 0.001) that was recorded over a 7 day period (Fig. 2). In contrast, acetamiprid was non-toxic (*P* = 0.298) even at the highest dose applied (µg/µl), but was related to extremely high variability in symptom development and bee survival for colonies rather than no toxicity. There was a significant colony effect [acetamiprid (*P* = 0.01), clothianidin (*P* = 0.038), imidacloprid (*P* = 0.003) and thiamethoxam (*P* = 0.025)] indicating that the amount of bee losses was related to the colony from which the bees were collected. There were a large proportion of bees that developed permanent late-onset neuromuscular dysfunction after exposure to clothianidin and thiamethoxam. These critical symptoms prevented the sustainability of life, and are normally included as an endpoint for survival assessments in vertebrate toxicity studies^34^. If we apply the same principle and replot the data to account for neuromuscular dysfunction (Fig. 2), the dose response curve moves downward. Pairwise comparisons of the means determined that clothianidin (*P* = 0.001), imidacloprid (*P* = 0.003) and thiamethoxam (*P* = 0.001), all caused significant reductions in bee survival at the lowest dose applied compared with untreated bees. The NOAEL was 37.5 ng/bee for acetamiprid. We could not calculate the NOAEL for clothianidin, imidacloprid or thiamethoxam as the lowest dose applied caused significant reductions in bee survival. The relative toxicity of the neonicotinoids was: clothianidin, imidacloprid, thiamethoxam≫ acetamiprid. Assuming a daily consumption rate of 120 µl of contaminated food, clothianidin, imidacloprid and thiamethoxam occur in sufficient quantities in natural bee food to have adverse effects on bumble bees.

A common early-onset symptom recorded for all neonicotinoids in the oral chronic toxicity studies was a curled abdomen with continuous muscular contractions between day 1 and 4. The early (1–4 days) - and late-onset (5–7 days) symptoms recorded for each neonicotinoid were: thiamethoxam caused early-onset hyperactivity leading to late-onset ataxia (lack of coordinated muscle movements for walking or standing) and the bees developed the appearance of a spider; acetamiprid/imidacloprid caused early-onset slow to no movements followed by late-onset ataxia and the bees developed the appearance of a spider; clothianidin caused hyperactivity followed by ataxia and the bees developed the appearance of a spider. Such time-based manifestations of poisoning are not new for pesticide exposure with invertebrates^36, 37^, but are for bee toxicology studies.

### Experiment 3. Acute exposure in summer honey bees

**Figure 3:**
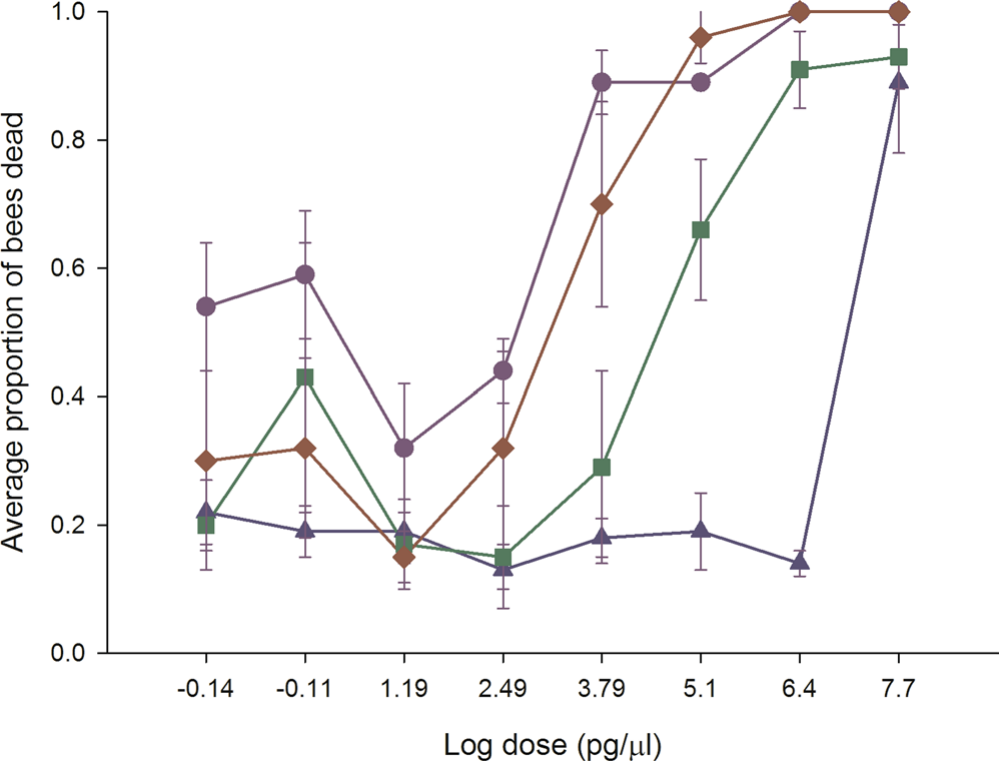
Clothianidin and thiamethoxam elicit biphasic dose-response curves in summer honey bees. Honey bees were provided untreated honey-water or honey- water containing 50 µg/µl, 2.5 µg/µl, 125 ng/µl, 6.25 ng/µl, 312.5 pg/µl, 15.6 pg/µl, 0.78 pg/µl and 0.039 pg/µl acetamiprid, imidacloprid, thiamethoxam and clothianidin for 24 hours in cage trials. The cages were then provided honey water ab libitum for 14 days. As a conservative estimate, all calculations were done using the highest consumption rate or 66 µl. Cages were assessed daily for the number of bees dead in a cage, the number of bees exhibiting symptoms, the type of symptoms, and the duration or outcome of the symptoms. The average values and standard errors are provided for each concentration applied. The data were collected from 6 independent experiments and expressed as proportion of bees dead. The four neonicotinoids are highlighted as follows: acetamiprid (blue triangle), clothianidin (red diamond), imidacloprid (green square) and thiamethoxam (purple circle).

Oral acute toxicity studies with acetamiprid (χ^2^ = 85.68, df = 8, *P* < 0.01) and imidacloprid (χ^2^ = 55.31, df = 8, *P* < 0.01) resulted in monotonic dose-response curves for honey bees (Fig. 3). The LD_50_ values were: 9.1 µg/bee acetamiprid and 32.8 ng/bee imidacloprid suggesting that neither neonicotinoid will adversely affect honey bees through ingestion of natural food. However, it is possible for honey bees to encounter sufficient quantities of imidacloprid in dusts created during the sowing of coated seeds to achieve adverse effects. In contrast, clothianidin (χ^2^ = 0.000308, df = 8, *P* < 0.01) and thiamethoxam (χ^2^ = 0.000106, df = 8, *P* < 0.01) exposure resulted in biphasic dose-response curves illustrating an EDC-like response. The curves are U-shaped and were most pronounced with thiamethoxam, with an average mortality of 72% with 6 pM to 133 pM (0.039–0.78 pg/µl), 88% with 3 nM to 53 nM (15.6–312.5 pg/µl) and 100% at 1 µM (1.1 pg/µl). Low levels of mortality were recorded within the OECD guidelines of 96 hr, but mortality peaked between 8 to 10 days post-challenge compared with untreated bees. There was a significant colony effect (P < 0.05) indicating that the amount of bee losses was related to the colony from which the bees were collected. Early- and late-onset symptoms were equivalent to those recorded for bumble bees, except that imidacloprid also caused the honey bees to line up in perfect rows or clusters. Unlike bumble bees, the honey bees displaying late-onset symptoms all succumbed 8 to 10 days post challenge. These results recommend the extension of the OECD guideline to 10 days to capture adverse effects on honey bees.

The NOAEL were 1 ng/bee for both acetamiprid and imidacloprid. The NOAEL could not be calculated for thiamethoxam or clothianidin as the bees exposed to the lowest dose displayed a significantly higher mortality than the untreated bees. Their LD_50_ values were: 9.1 µg/bee acetamiprid and 32.8 ng/bee imidacloprid. A single LD_50_ value could not be generated for clothianidin and thiamethoxam as there were two peaks associated with bee mortality. The LD_50_ values were: 2.6 pg/bee and 36.7 pg/bee for clothianidin; 34.7 pg/bee and 51.4 pg/bee for thiamethoxam. The relative toxicity of the neonicotinoids was: clothianidin > thiamethoxam≫  imidacloprid≫ acetamiprid. Assuming a daily consumption rate of 66 µl of contaminated food, clothianidin and thiamethoxam occur in sufficient quantities in natural bee food to have adverse effects on honey bees.

### Experiment 4. Chronic exposure in summer honey bees

**Figure 4:**
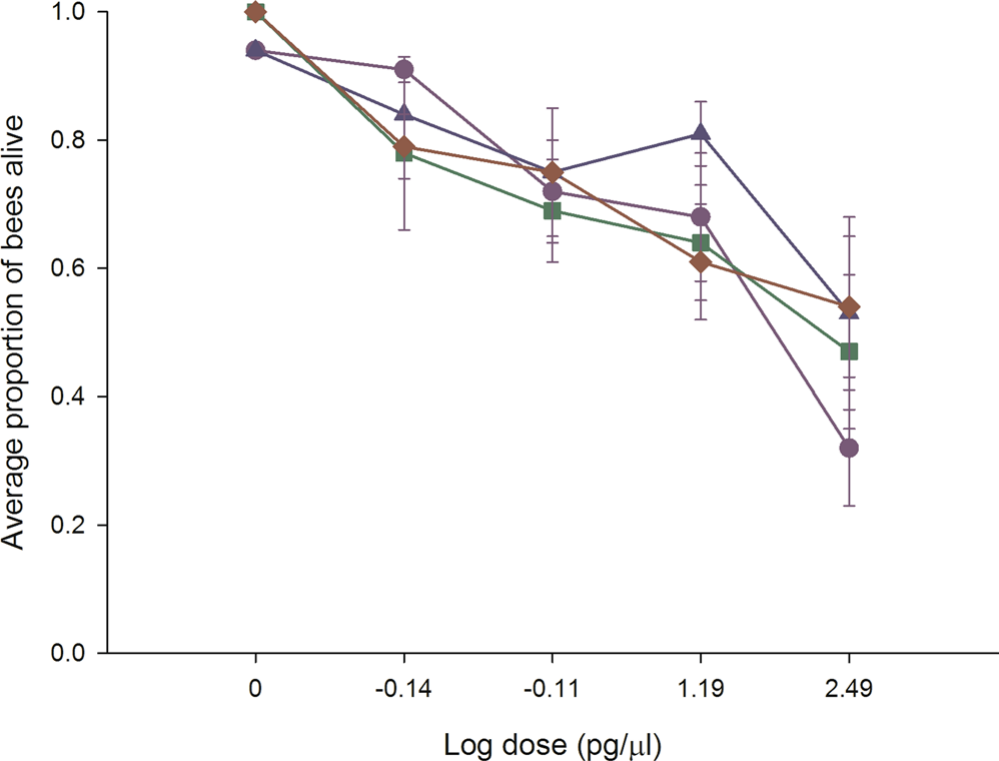
Neonicotinoids slightly decrease summer honey bee survival at field relevant levels. Honey bees were provided untreated honey-water or honey- water containing 312.5 pg/µl, 15.6 pg/µl, 0.78 pg/µl and 0.039 pg/µl acetamiprid, clothianidin, imidacloprid and thiamethoxam for 14 days in cage trials. As a conservative estimate, all calculations were done using the highest consumption rate or 66 µl. Cages were assessed daily for the number of bees alive in a cage, the number of bees exhibiting symptoms, the type of symptoms, and the duration or outcome of the symptoms. The average values and standard errors are provided for each concentration applied. The data were collected from 6 independent experiments and expressed as the proportion of bees alive. The four neonicotinoids are highlighted as follows: acetamiprid (blue triangle), clothianidin (red diamond), imidacloprid (green square) and thiamethoxam (purple circle).

In oral chronic toxicity studies, continuous neonicotinoid exposure over a 10-day period resulted in a dose-dependent decline in honey bee survival for acetamiprid (*P* = 0.044), clothianidin (*P* = 0.013), imidacloprid (*P* = 0.032) and thiamethoxam (*P* = 0.001; Fig. 4). There was a significant colony effect for the bee responses to imidacloprid (*P* = 0.022), but not with the other neonicotinoids. Pairwise comparisons of the means showed that increasing doses of clothianidin (*P* = 0.013), imidacloprid (*P* = 0.032) and thiamethoxam (*P* = 0.001), were associated with decreasing bee survival, but only imidacloprid had this effect at field-realistic concentrations recorded for food^25^. The NOAEL were 1 ng/bee for acetamiprid, 0.051ng/bee for clothianidin, 1 ng/bee for imidacloprid and 0.051ng/bee for thiamethoxam. The relative toxicity of the neonicotinoids was: clothianidin, thiamethoxam > acetamiprid, imidacloprid. Honey bees displayed the same early- and late-onset symptoms as bumble bees, but affected bees succumbed by day 10. Therefore, adverse effects are captured without the necessity to include time-based neurological symptoms of poisoning. Assuming a daily consumption rate of 66 µl of contaminated food, imidacloprid occurs in sufficient quantities in natural bee food to have adverse effects on honey bees.

### Experiment 5. Acute exposure in winter honey bees

Worker winter bees are about 6 months old and have undergone hormonal changes that facilitate overwintering when compared with the summer worker honey bees. Using the data obtained over the sampling period, acute toxicity studies with acetamiprid (χ^2^ = 42.6, df = 8, *P* < 0.01), clothianidin (χ^2^ = 4.01, df = 8, *P* < 0.01), imidacloprid (χ^2^ = 40.2, df = 8, *P* < 0.01) and thiamethoxam (χ^2^ = 20.0, df = 8, *P* < 0.01) resulted in biphasic dose-response curves for winter honey bees during spring emergence. If we are conservative and use the data for the entire sampling period, a single LD_50_ value could not be generated for any of the neonicotinoids with winter honey bees, as there were two mortality peaks illustrating an EDC-like response. The LD_50_ values were: 21.5 ng /bee and 72.6 µg/bee for acetamiprid; 0.013 pg/bee and 6.36 pg/bee for clothianidin; 29.9 pg/bee and 1.4 µg/bee for imidacloprid; 0.02 pg/bee and 2.9 ng/bee for thiamethoxam. In early spring, which is when the bees are the most sensitive, LD_50_ and NOAEL values could not be generated for thiamethoxam or clothianidin due to high mortality present at all applied doses. The relative toxicity of the neonicotinoids was: clothianidin, thiamethoxam > imidacloprid > acetamiprid. Assuming a daily consumption rate of 66 µl of contaminated food, clothianidin, imidacloprid and thiamethoxam occur in sufficient quantities in natural bee food to have adverse effects on winter honey bees in spring.

**Figure 5:**
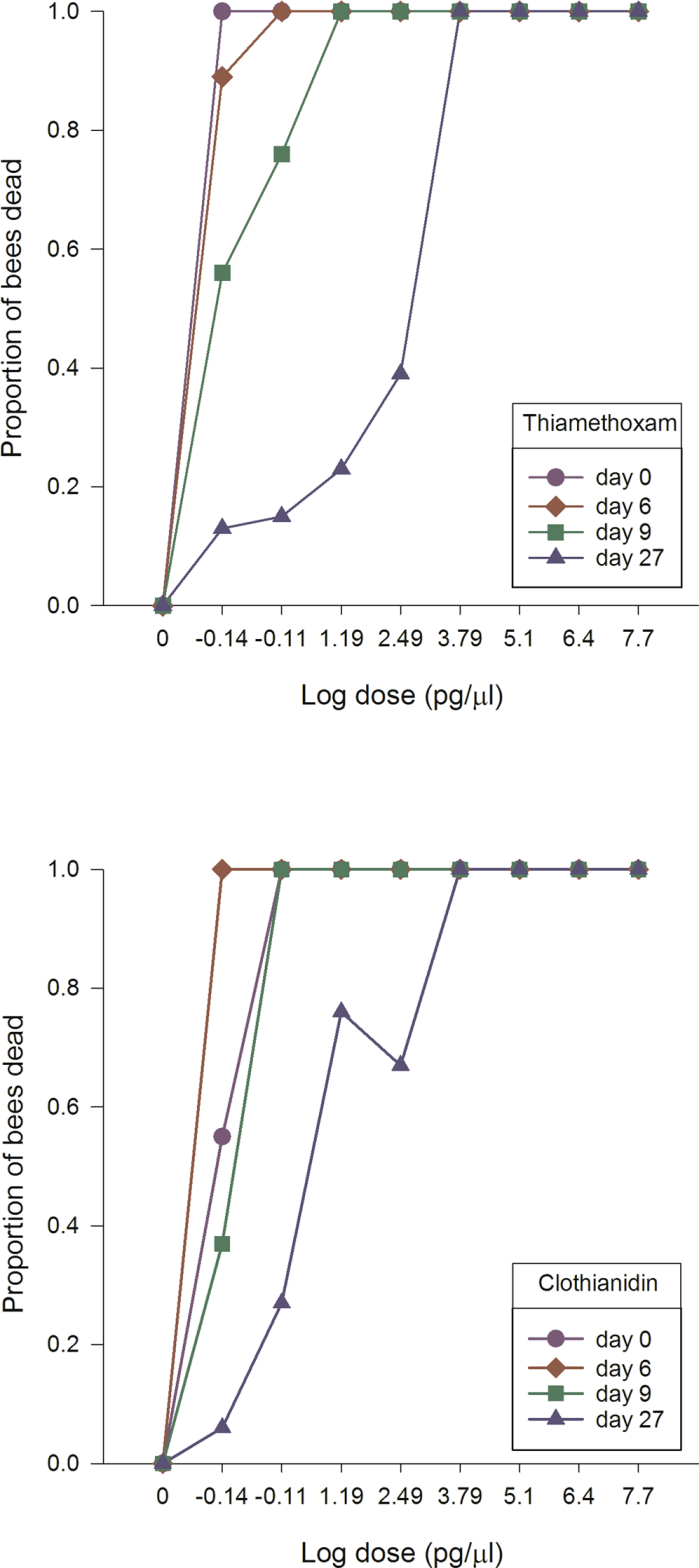
Clothianidin and thiamethoxam highly increase winter honey bee mortality in spring. Winter worker bees were provided untreated honey-water or honey-water containing 50 µg/µl, 2.5 µg/µl, 125 ng/µl, 6.25 ng/µl, 312.5 pg/µl, 15.6 pg/µl, 0.78 pg/µl and 0.039 pg/µl acetamiprid, imidacloprid, thiamethoxam and clothianidin for 6 hours in cage trials. The cages were then provided honey water ab libitum for 14 days. We used the consumption rate of 54 µl for all LD_50_ calculations. Cages were assessed daily for the number of bees dead in a cage, the number of bees exhibiting symptoms, the type of symptoms, and the duration or outcome of symptoms. The average values and standard errors are provided for each concentration applied. The data were collected from 6 independent experiments and expressed as proportion of bees dead. Results are provided for each week during the monitoring of the winter bee response.

**Figure 6:**
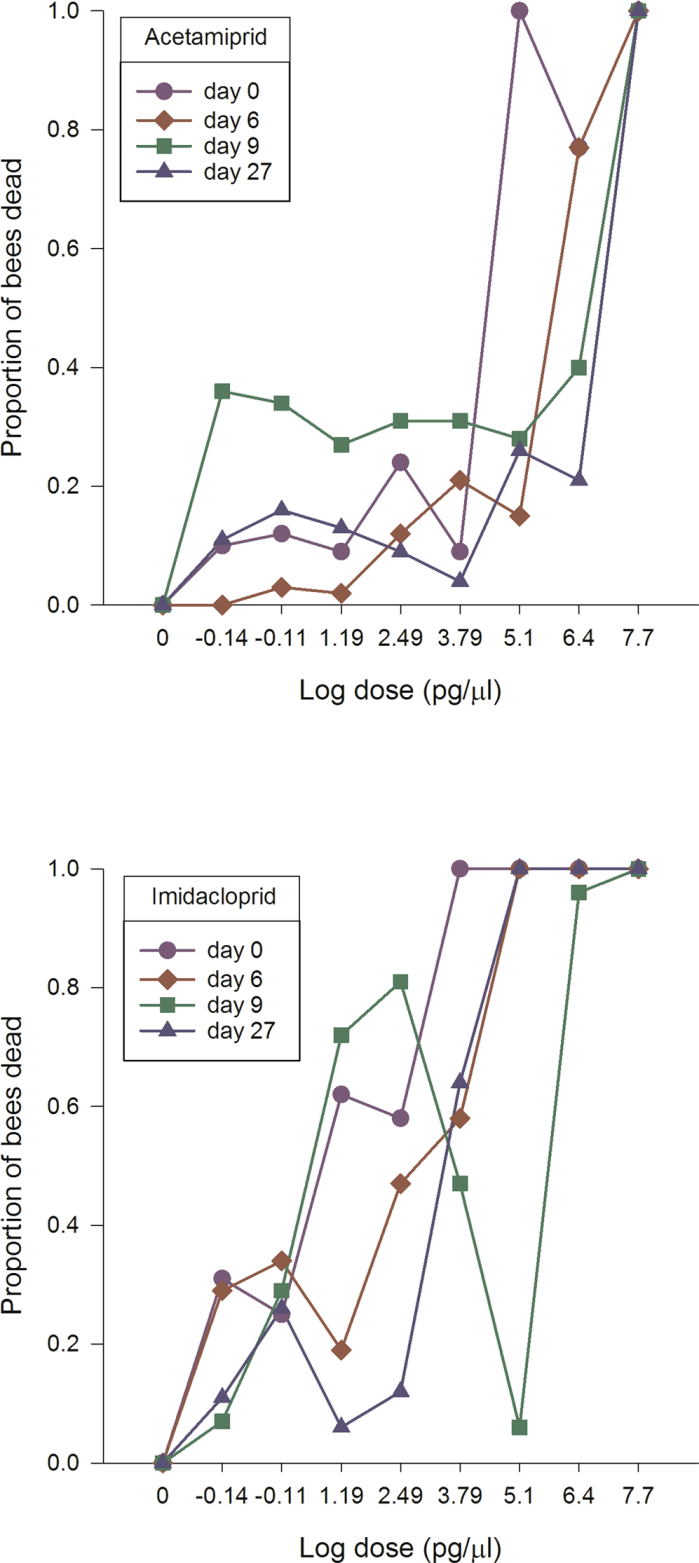
Imidacloprid and acetamiprid increase winter honey bee mortality in spring. Winter worker bees were provided untreated honey-water or honey-water containing 50 µg/µl, 2.5 µg/µl, 125 ng/µl, 6.25 ng/µl, 312.5 pg/µl, 15.6 pg/µl, 0.78 pg/µl and 0.039 pg/µl acetamiprid, imidacloprid, thiamethoxam and clothianidin for 6 hours in cage trials. The cages were then provided honey water ab libitum for 14 days. We used the consumption rate of 54 µl for all LD_50_ calculations. Cages were assessed daily for the number of bees dead in a cage, the number of bees exhibiting symptoms, the type of symptoms, and the duration or outcome of symptoms. The average values and standard errors are provided for each concentration applied. The data were collected from 6 independent experiments and expressed as proportion of bees dead. Results are provided for each week during the monitoring of the winter bee response.

The winter honey bees displayed different sensitivity patterns than summer-collected honey bees after neonicotinoid exposure. Thiamethoxam displayed a flat dose response curve representing 100% mortality, regardless of dose in early spring (Fig. 5). In late spring or after 20 days, thiamethoxam sensitivity started to decline at the lowest doses achieving 80% and 55% mortality, respectively. After 27 days, the dose-response curve resembled that for honey bees in the summer. Clothianidin followed the same pattern as thiamethoxam except in early spring where the lowest dose was only associated with about 50% mortality (Fig. 5). In contrast, imidacloprid caused 100% mortality for the 50 µg/µl–312.5 pg/µl doses, high mortality (~80%) for the 15.6–0.78 pg/µl doses, and low mortality for the 0.039 pg/µl dose (Fig. 6). After 8 days, the dose response curve changed into a biphasic dose-response curve, with a mortality peak (72–100%) at unrealistic field concentrations and another peak at field-realistic concentrations. After 20 days, the imidacloprid dose-response curve for winter honey bees resembled the dose-response curve for newly-emerged worker honey bees. Acetamiprid followed the same pattern as imidacloprid except high toxicity was only recorded at unrealistic field concentrations (Fig. 6).

The early-onset symptoms for winter bees developed quickly (minutes) at all doses of thiamethoxam and clothianidin. In contrast, newly-emerged worker honey bees developed early-onset symptoms over a longer time frame (hours to days) and at only a few high doses. In the first week, the early-onset symptoms included vomiting, salivation, abdominal curl, abdominal contractions, tremors and paralysis that are suggestive of overstimulation of mAChR/nAChR in the nervous system. Paralysis was evident within minutes at the higher four doses of thiamethoxam and clothianidin compared with untreated bees, while the other doses caused complete paralysis within 1 hr. There was no recovery of any bees exhibiting these symptoms. By the fourth week, paralysis was evident at the higher four doses (4 hr), but was replaced by neuromuscular dysfunction at the lower four doses (5–8 days). EDC actions are affected by developmental stage and temperature in other invertebrate species, and it would appear that these factors also are contributing to winter honey bee susceptibility to neonicotinoids.

In order to understand the underlying mechanism for rapid-onset of symptoms, we compared the gross anatomy of winter honey bees collected in the first two weeks of March with those collected in the last two weeks of March. We examined a number of tissues, and although tissues such as the fat body were larger than summer honey bees, the changes to the midgut were obvious. All winter honey bees collected in the first three weeks lacked a regionalization or striation pattern that is characteristic of a functional midgut. The tissue was also considerably smaller and narrower, about half the width of a midgut from summer honey bees. We did not observe any columnar cells in the midguts collected in the first 2 1/2 weeks when examined at 400 times magnification using an inverted microscope, but these cells were evident in midguts examined thereafter. In the third week, we collected bees from a colony housed in a controlled environment building (5 °C) that were not provided access to the outside environment. These midguts matched the midguts from winter bees collected from the bee yard early in March. Therefore, the faster appearance of acute symptoms and the broader dose range achieving high toxicity in winter honey bees suggests an enhanced neonicotinoid bioavailability likely through greater gut-transfer after oral ingestion.

### Experiment 6. Acute exposure in leafcutter bees

**Figure 7:**
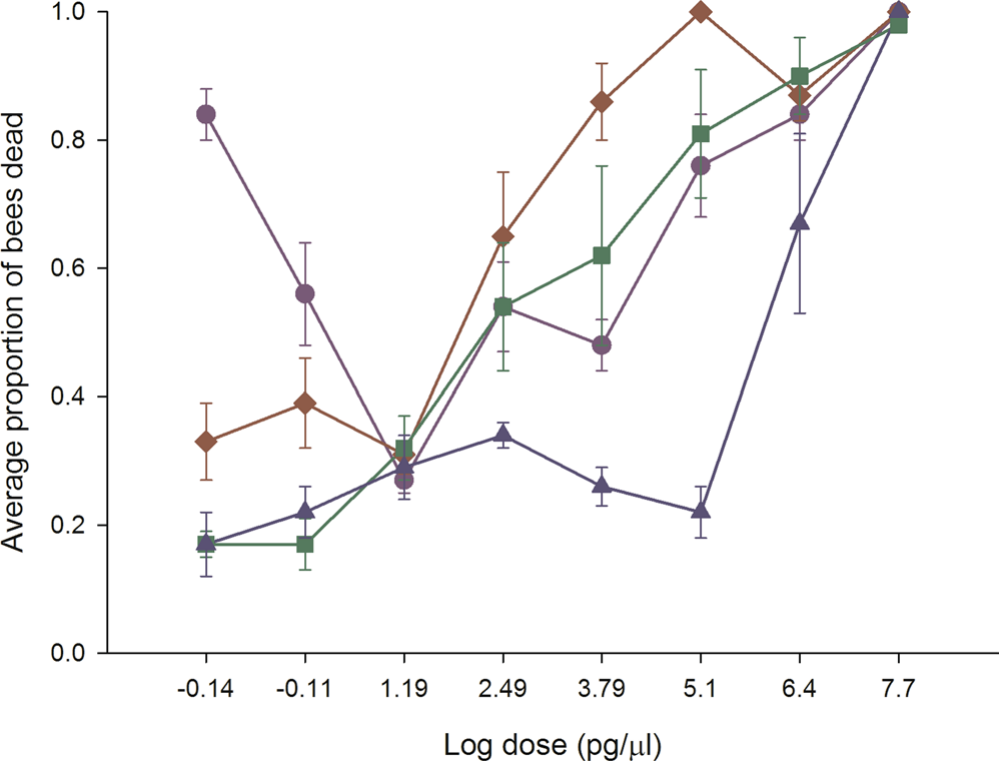
Clothianidin, imidacloprid and thiamethoxam elicit biphasic dose-response curves in leafcutter bees. Leafcutter bees were provided untreated honey-water or honey- water containing 50 µg/µl, 2.5 µg/µl, 125 ng/µl, 6.25 ng/µl, 312.5 pg/µl, 15.6 pg/µl, 0.78 pg/µl and 0.039 pg/µl acetamiprid, imidacloprid, thiamethoxam and clothianidin for 24 hours in cage trials. The cages were then provided honey-water ab libitum for 14 days. As a conservative estimate, we used the highest consumption rate or 34 µl for all LD_50_ calculations. Cages were assessed daily for the number of bees dead in a cage, the number of bees exhibiting symptoms, the type of symptoms, and the duration or outcome of symptoms. The average values and standard errors are provided for each concentration applied. The data were collected from 6 independent experiments and expressed as proportion of bees dead. The four neonicotinoids are identified as follows: acetamiprid (blue triangle), clothianidin (red diamond), imidacloprid (green square) and thiamethoxam (purple circle).

Acute toxicity studies with acetamiprid (χ^2^ = 58.7, df = 8, *P* < 0.01) resulted in a monotonic dose-response curve for newly-emerged leafcutter bees (Fig. 7). This neonicotinoid was non-toxic at field realistic concentrations. In contrast, clothianidin (χ^2^ = 39.5, df = 8, *P* < 0.01), imidacloprid (χ^2^ = 40.29, df = 8, *P* < 0.01) and thiamethoxam (χ^2^ = 152.84, df = 8, *P* < 0.01) exposure resulted in biphasic dose-response curves with leafcutter bees illustrating an EDC-like response. The curves are U-shaped and were most pronounced with thiamethoxam, with an average mortality of 69% for 6 pM to 133 pM (0.039–0.78 pg/µl), 43% for 3 nM–1.1 µM (15.6 pg/µl–6.25 ng/µl) and 100% for 8.55 mM (50 µg/µl). There was a significant effect of area (P < 0.05) indicating that bee sensitivity was related to the area from which the solitary bees were collected. Low levels of mortality were recorded within the OECD guidelines of 96 hr, but mortality peaked between 8 to 10 days post-challenge compared with untreated bees. Early- and late-onset symptoms were equivalent to those recorded for bumble bees. There was one new late-onset symptom unique to newly-emerged leafcutter bees, which was that “no buzz” was detected for adults exposed to clothianidin. Leafcutter male and female bees normally vibrate when picked-up with tweezers creating a buzz. Since these bees buzz-pollinate, this could potentially alter pollination efficiencies in crops. As with the honey bees, symptomatic leafcutter bees succumbed by day 10. These results recommend the extension of the OECD guideline to 10 days to capture adverse effects on leafcutter bees.

The NOAEL were 4.2 µg/bee for acetamiprid, 26 pg/bee imidacloprid and 26 pg/bee clothianidin. We could not calculate a NOAEL for thiamethoxam as the lowest dose applied was associated with significantly greater mortality than untreated bees. The LD_50_ value was 9.3 µg/bee for acetamiprid and is equivalent to about the third highest dose applied. A single LD_50_ value could not be generated for clothianidin, imidacloprid and thiamethoxam as there were two peaks associated with bee mortality. The LD_50_ values were: 0.0006 pg/bee and 14.1 pg/bee for clothianidin; 3.2 pg/bee and 6.1 ng/bee for imidacloprid; 5 pg/bee and 98 pg/bee for thiamethoxam. The relative toxicity of the neonicotinoids was: clothianidin≫ imidacloprid, thiamethoxam≫ acetamiprid. Assuming a daily consumption rate of 34 µl of contaminated food, clothianidin, imidacloprid and thiamethoxam occur in sufficient quantities in natural bee food to have adverse effects on leafcutter bees.

### Experiment 7. Chronic exposure in leafcutter bees

**Figure 8:**
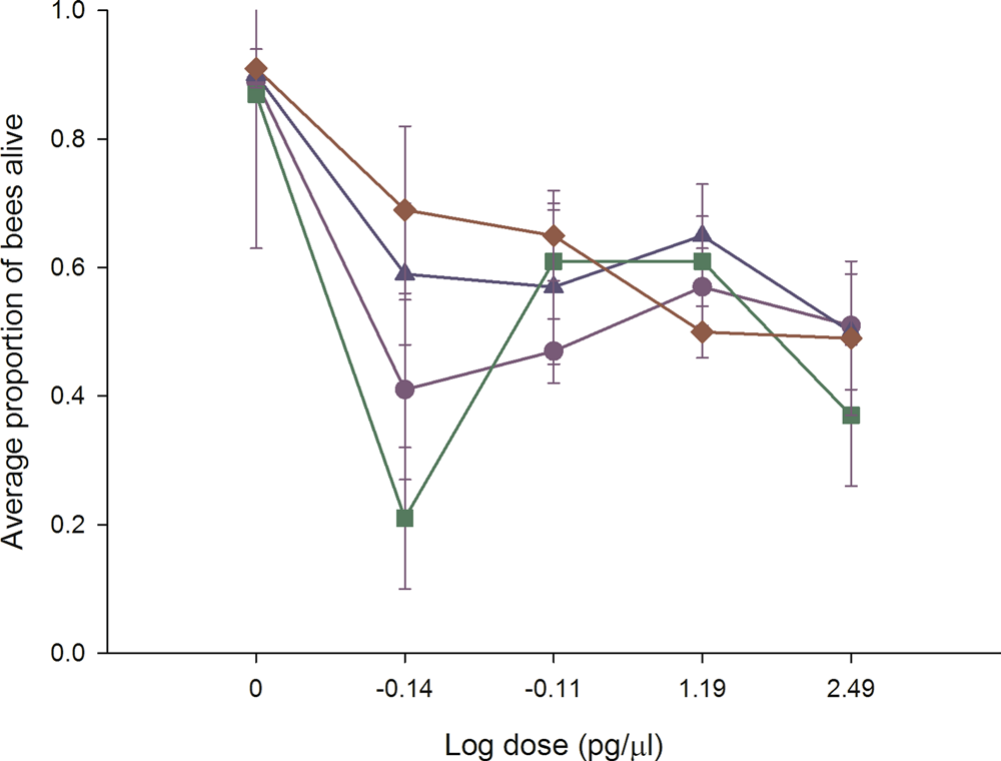
Neonicotinoids decrease leafcutter bee survival at field relevant levels. Leafcutter bees were provided untreated honey-water or honey- water containing 312.5 pg/µl, 15.6 pg/µl, 0.78 pg/µl and 0.039 pg/µl acetamiprid, imidacloprid, thiamethoxam and clothianidin for 14 days in cage trials. As a conservative estimate, all calculations were done using the highest consumption rate or 34 µl. Cages were assessed daily for the number of bees alive in a cage, the number of bees exhibiting symptoms, the type of symptoms, and the duration or outcome of the symptoms. The average values and standard errors are provided for each concentration applied. The data were collected from 6 independent experiments and expressed as proportion of bees dead. The four neonicotinoids are highlighted as follows: acetamiprid (blue triangle), clothianidin (red diamond), imidacloprid (green square) and thiamethoxam (purple circle).

In oral chronic toxicity studies, continuous neonicotinoid exposure resulted in a dose-dependent decline in survival for acetamiprid (*P* = 0.006), clothianidin (*P* = 0.04), imidacloprid (*P* = 0.001) and thiamethoxam (*P* = 0.04) that was recorded over a 10 day period (Fig. 8). There was a significant effect of area (*P* < 0.01) indicating that the amount of bee losses was related to the area from which the bees were collected. The NOAEL could not be generated for any of the neonicotinoids as the lowest dose applied was associated with significantly lower survival than untreated bees. The relative toxicity of the neonicotinoids was: acetamiprid, imidacloprid, thiamethoxam > clothianidin. Leafcutter bees displayed the same early- and late-onset symptoms as bumble bees except for the “no buzz”, but succumbed by day 10. Therefore, adverse effects are captured without the necessity to include time-based neurological symptoms of poisoning. Assuming a daily consumption rate of 34 µl of contaminated food, all four neonicotinoids occur in sufficient quantities in natural bee food to have adverse effects on leafcutter bees.

## Discussion

This study expanded the dose range applied in oral acute and chronic toxicity studies to include field realistic levels encountered by bees in their stored food such as pollen and honey^25, 26^. By doing so, we identified a new feature of adverse effects and neonicotinoid dose for bumble bees, honey bees, and leafcutter bees which are manifested by biphasic dose-response curves. These types of curves provide two LD_50_ values, instead of the single LD_50_ value reported to date for neonicotinoid exposure and bees. For all three bee species, one of the LD_50_ values falls within the dose range found in stored food for clothianidin and thiamethoxam, but this only occurred with imidacloprid for bumble bees and leafcutter bees. This study also expanded the time frame for assessing neurological symptoms in bees to determine whether earlier studies had missed time-based neurological symptoms, and they had. A ten-day assessment period was required to capture adverse effects on bees in acute toxicity studies with worker honey bees, compared with 4 days used in all earlier neonicotinoid studies. The same time period was also required for leafcutter bees, but a shorter time period, 7 days, was adequate for bumble bees. We confirmed early-onset symptoms associated with neonicotinoid intoxication with all three bee species including paralysis, tremors, excessive grooming, proboscis extension, salivation, vomiting, hyperactivity, slow to no movement, and no righting behavior. Generally, the dose at which these symptoms occurred was above levels detected in food^25^, but could be achieved with clothianidin and thiamethoxam dusts^10^. The previously unknown late-onset symptoms were neuromuscular in nature or peripheral neuropathies that were visually assessed as periodic hyperactivity, “lost” orientation, and ataxia in all three bee species. The concentrations at which these intoxications occurred was at levels present in their food^25^. One key difference among the three bee species was the time required to proceed from neuromuscular dysfunction to death. Impaired bumble bees were alive at the end of the assessment period (14 days), which would cause an underestimation of mortality in acute toxicity studies and overestimation of survival in chronic toxicity studies. Because bumble bees didn’t recover until their death, this study recommends the application of neuromuscular dysfunction as a non-lethal endpoint in toxicity studies with worker bumble bees. In contrast, honey bees and leafcutter bees with ataxia succumbed by day 10, making the inclusion of neuromuscular dysfunction unnecessary if the assessment period is extended from 4 to 10 days.

Evaluation of neonicotinoid risk for bees in agroecosystems requires an understanding of field-realistic levels encountered in bee food^25–27^. There is large variation in the concentration and type of neonicotinoids present in pollen, honey and worker honey bees which reflects contact with contaminated crops, weeds, seed dusts and soil^27^. In western Canada, clothianidin (detected in 68%of samples) and thiamethoxam (75% of samples) are the most frequently detected neonicotinoids in honey samples at mean concentrations of 8.2 ng/g and 17.2 ng/g, respectively^25^. In other areas, concentrations of clothianidin in honey are much lower, 0.9 ng/g^25, 26, 28^. Analysis of healthy and impaired worker honey bees have also been examined with the goal of understanding how much neonicotinoid exposure is tolerated. For example, healthy worker honey bees have non-detectable to 2.9 ng/g clothianidin present in their bodies, while affected bees have 3.8–13.3 ng/g clothianidin present^25^. Similarly, pollen collected by foraging worker honey bees have higher concentrations of clothianidin in spring than in summer^27^ suggesting a seasonality to exposure. Previous acute toxicity studies with neonicotinoids applied to worker honey bees did not include the concentrations found in pollen and honey^19–22^. In our study, expanding the dose range to encompass field realistic doses and increasing the assessment period has provided evidence for a potential risk to nurse-aged worker honey bees in a single visit to a contaminated food source such as stored pollen^46^, which is more likely to contain significant neonicotinoid concentrations in Canadian agroecosystems^25^. For example, a recent oral acute toxicity study with mainly forager-aged worker honey bees provided LD_50_ values for analytical grade neonicotinoids: 18 ng/bee for clothianidin, 116 ng/bee for imidacloprid and 11.8 ng/bee for thiamethoxam^26^. However, there is no data available for nurse-aged worker honey bees, and herein, we determined that the LD_50_ value for nurse-aged worker honey bees was three times lower for imidacloprid compared with forager-aged worker honey bees and are about a thousand times lower for clothianidin and thiamethoxam. The same level of sensitivity to clothianidin and thiamethoxam were also found with newly-emerged leafcutter bees and bumble bees suggesting that part of the issue with assessing neonicotinoid risk and bee health has arisen from the use of forager-aged worker honey bees which are not representative of neonicotinoid risk to other honey bee adult stages or other bee species.

Chronic toxicity studies are undertaken to monitor the development of adverse effects as a result of long term or repeated exposures to insecticides. Early oral chronic toxicity studies applying imidacloprid (12 µg/kg or 1.4 ng/day) with forager-aged worker honey bees identified impairment of associative learning^29–33^. Similarly, applying imidacloprid (0.020 mg/kg or 2.4 ng/bee) to honey bee colonies did not significantly change mortality, feeding activity, wax comb production, reproduction or colony performance^28^. These results together with contaminated plant studies^26^ provided evidence that honey bee colonies were unlikely to be adversely affected by imidacloprid in agroecosystems. For well over a decade, managed honey bee colonies continued to decline worldwide^1–3^, providing the impetus to reevaluate chronic toxicity for neonicotinoids using field-realistic doses. Honeybee colonies chronically exposed to thiamethoxam (5 ppb or 0.005 ng/µl) and clothianidin (2 ppb or 0.002 ng/µl) contaminated food resulted in declining numbers of adult bees (28% less), brood (13% less), honey production (29% less) and pollen collections (19% less)^38^. These colonies recovered from the exposure and overwintered successfully, but there was a significant slower growth of neonicotinoid-exposed colonies in the spring that was linked to queen failure. From our studies, both of these neonicotinoids significantly reduce nurse-aged worker honey bee survival providing an explanation for the quantified losses in this colony-level study. In another study, applying 10–100 ppb (0.01–0.1 ng/µl or 0.66 ng/day–6.6 ng/day) imidacloprid in sugar-syrup provided to small-sized honey bee colonies resulted in significant reductions in: egg-laying and locomotor activity by queens, foraging and hygienic activities of worker honey bees; brood production; and pollen stores^8^. We were not able to confirm these findings, but we did determine that nurse-aged worker honey bees, chronically exposed to the highest dose applied in the chronic toxicity studies would contribute to the recorded decrease in worker honey bee activities and survival. We conclude that clothianidin, imidacloprid and thiamethoxam occur in sufficient quantities within honey bee food stores to adversely affect vulnerable bee stages^41^ after single or repeated ingestion of contaminated food stores.

In bumble bee colonies, oral chronic toxicity studies applying 4.8 ng per day imidacloprid (Gaucho) in syrup or pollen resulted in a significant low-level reduction in worker *B. terrestris* survival (10%), but not in other colony health parameters^25^. It was concluded that *B. terrestris* colonies were unlikely to be adversely affected by long-term exposure. More recent oral chronic toxicity studies examined the impact of field-realistic concentrations (10, 20, 50, and 100 ppb; 1 ppb = 0.001 ng/µl) of imidacloprid and clothianidin on colony health using caged queenright colonies of *B. impatiens* ^39^. Both neonicotinoids applied at 20 ppb caused a significant reduction in queen survival, worker movement, colony consumption, and colony weight compared to untreated colonies. There was also a reduction in the number of worker bumble bees collecting syrup and bringing it back to the nest. Affected worker bumble bees also were observed as sitting immobilized for weeks on the floor of the flight box. The significance of this change in worker bumble bee movement was not discussed with respect to the endpoints measured in that study. Our studies determined that worker bumble bees develop neuromuscular dysfunction at the lowest dose applied in the chronic toxicity studies with clothianidin and imidacloprid. By following the progression of symptoms in the worker bumble bees, we determined that affected bees do not recover and moreover did not go to the feeder even at 14 days post-challenge. After this time, all bees succumbed by day 21making this a valid non-lethal endpoint for survival or mortality estimates. Therefore, our results provide an explanation for the earlier reported lower numbers of foraging worker bees, lower numbers of moving bees, lower colony consumption and lower colony weight. In another chronic toxicity study examining learning performance with trainable worker bumble bees, control bees learnt the task faster than bees receiving 2.4 ppb (27% faster) and 10 ppb (38% faster) thiamethoxam in a sugar-syrup^33^. Similar concentrations in our chronic toxicity studies were associated with neuromuscular dysfunction (30% bees), and by inference, clothianidin and imidacloprid likely also affect learning performance. Of note, these behavioral studies are performed using mature worker bumble bees within hours of receiving a neonicotinoid dose, potentially underestimating neurological impairment.

There is only one contact acute toxicity study with imidacloprid applied to adult leafcutter bees. It concluded that imidacloprid was non-toxic^6^. We performed comprehensive acute and chronic toxicity studies with newly-emerged leafcutter bees and determined that this species was more sensitive to neonicotinoids than worker honey bees. Interestingly, the leafcutter bee responses to neonicotinoid exposure closely resembled those manifested by bumble bees. For example, acetamiprid was toxic to leafcutter bees and mature bumble bees, but not honey bees in the chronic toxicity studies. This suggests that bumble bees and leafcutter bees may not metabolize neonicotinoids in the same manner as worker honey bees, where acetamiprid is rapidly degraded to non-toxic components^4, 5^. We conclude that acetamiprid, clothianidin, imidacloprid and thiamethoxam occur in sufficient quantities within natural foods they collect from the field to adversely affect leafcutter bees.

Larvicides and adulticides are insecticides that are developed for insect pests to address age-specific differences in susceptibility^36, 47^. In the current study, worker honey bees and leafcutter bees were collected at adult eclosion which encompasses the emergence of the insect from the pupal cell followed by expansion and hardening of the wings. All phases of this process represent an orchestrated hormonal interplay leading to what we know as the adult bee^48–50^. Prior to eclosion, midgut tissues have little structure, but become regionalized after adult emergence, remaining stable except when recovering from acute tissue damage^49^. There are reviews on the outcome of insecticide exposure in target pest insects^5, 47^, but as bees are non-target arthropods, less information is available regarding their stage-specific susceptibility. Our data support a greater sensitivity of newly-emerged nurse-aged worker honey bees for imidacloprid exposure compared with forager-aged worker honey bees which is likely related to the changes in midgut permeability associated with the regionalization process. To better understand how developmental status could influence bee susceptibility to neonicotinoids, we investigated another hormone-modulated adult stage in the honey bee life cycle, which are winter worker honey bees in spring. The transition from summer worker honey bee to a winter worker honey bee in fall involves changes in juvenile hormone and neurotransmitter levels that together reduce winter bee activities^45, 50^. In spring, this process is inverted. Past studies examining the impact of neonicotinoids on winter honey bee survival either did not measure any adverse effects on bee activities^28^ or winter honey bees abandoned the colonies and eventually died^51^. In the current study, for the first 14 days, there was a dose-dependent consumption rate for the summer honey bees but not for the winter honey bees. Instead, the winter honey bees consumed the entire volume of honey water within minutes which caused 100% bee mortality with thiamethoxam, regardless of dose, and 100% bee mortality with clothianidin at all doses except the lowest dose. In contrast, winter honey bees exposed to imidacloprid and acetamiprid displayed a dose-response curve that shifted to the left (higher sensitivity) relative to summer honey bees, but generally were not 100% toxic at the four lowest doses. More significantly, the winter bees exposed to thiamethoxam and clothianidin were intoxicated rapidly with paralysis detected within minutes of consuming the highest four doses and within an hour for the lowest four doses. The same rapid onset and severity of symptoms were recorded with imidacloprid and acetamiprid for any dose achieving high mortality. Intoxicated bees showed typical early-onset neurological symptoms described for pesticide poisoning. This supports the first few weeks following the end of overwintering in the spring as the most sensitive period identified to date for honey bees. We continued to perform weekly bioassays to see how the switchover from an inactive to an active winter honey bee would affect neonicotinoid susceptibility. The initial flat line dose-response curve for thiamethoxam and clothianidin corresponding to high sensitivity of winter bees transitioned to the less sensitive summer honey bee dose-response curve after twenty-three days or by late spring. To test the hypothesis that leaving the colony and being exposed to environmental triggers, such as temperature or food, were essential to the reduced toxicity, we examined whether winter honey bees that were contained in an environmentally-controlled building in late spring, had the same susceptibility as bees collected from outside colonies in early spring, and they did. The rapid onset and the presence of severe neurological symptoms i﻿n early spring suggested that neonicotinoids were either transferred unimpeded through the midgut or there were a higher density of target receptors than in late spring. Our study documented a change in midgut structure that closely followed winter honey bee sensitivity to the neonicotinoids. However, target neuronal receptors are also known to dramatically decline in fall for winter honey bees making them less susceptible to pesticides^46^ and it is plausible that this process is inverted in spring. We conclude that midgut barrier function and receptor density may be a factor affecting neonicotinoid toxicity in winter honey bees in spring, and this feature is shared with all overwintered adult bees. There are very few studies recording the toxicity of neonicotinoids to hormonally-sensitive stages such as the winter worker honey bee. In one example, imidacloprid-treated honey bee colonies that contained a low number or 1500 bees showed an inconsistent number of larvae and a decrease in the number of pupae that correlated with increasing dose^8^. Similarly in larger 3000-honey bee colonies, 100 ppb imidacloprid reduced the number of larvae and pupae. Further studies with immature bee stages are needed to elucidate the influence of hormonal status on neonicotinoid sensitivity.

If we combine semi-field and field outcomes for neonicotinoid exposure with our results from the acute and chronic studies with the three bee species, it is possible to predict exposure-outcomes for managed and wild bee populations. For managed social honey bees, winter bee losses may be seen as lower spring cluster size and this in turn, would reduce the size of the spring first brood or cause complete colony losses in spring. Once the first generation of new bees is established, then the neonicotinoid exposure for this and subsequent generations is associated with ingestion of contaminated food. For wild social bumble bees, exposure over several years could cause localized extinctions or large scale population losses. For example, bumble bees have an annual life cycle that ends with new queens that leave the nest and enter winter diapause. During this process, juvenile hormone levels decline and in spring this process is inverted^45^. Like the winter honey bees, emerging queens in spring likely are highly vulnerable to neonicotinoid exposure. Spring queen losses would directly reduce the number of nests formed in a season and over time result in lower populations. For wild solitary bees, there would also be great losses of emerging adults in spring from neonicotinoid exposure, leading to lower breeding populations. However, for managed solitary leafcutter bees, emergence is a very controlled process protecting the bees from any exposure during spring planting. We conclude that the winter honey bee and by inference other overwintering adult bees are likely sensitive to thiamethoxam and clothianidin exposure during spring emergence. To prevent these issues from affecting wild and managed bee populations, a bee emergence forecasting system could be used to predict when it should be safe to plant seeds coated with neonicotinoids.

As for target pest insects, neonicotinoids interact with nAChR in bees^4, 16^, and thiamethoxam also interacts with mAChR that occur in the supraoesophageal ganglion, abdominal ganglia, muscles and other innervated tissues^5, 16, 17^. There is evidence that interactions of agonists with mAChR can result in biphasic dose-response curves in isolated cervical ganglions of vertebrates^52^, potentially providing an indication of underlying mechanisms. In that study, muscarine activation of the M1 and M2 receptors resulted in a biphasic dose-response curve with the M1 response associated with depolarization events and the M2 response associated with hyperpolarization events. Evidence for multiple receptor targets is available for clothianidin which induces a biphasic effect on pheromone-guided behavior suggestive of dual receptor targets in worker honey bees^31^. Another explanation for the nature of the dose-response curve may come from the steps involved in transferring the oral dose from the midgut lumen to the haemocoel, which is an open circulatory system. After ingestion, transfer of insecticide to the haemocoel will be dependent upon movement across the anterior midgut, and whether any receptors are located within the midgut tissue. Once transferred, the neonicotinoids would rapidly move from the abdomen to the head region as part of the normal movements of the dorsal vessel or heart. Any neonicotinoid having a mode of action that facilitates entry would remove the first barrier to transfer, the midgut. Thiamethoxam is cytotoxic and enhances cell replication in vertebrate systems^53^ and it damages the midgut of worker honey bees in the first few days after ingestion of an efficacious dose causing downstream damage to the insect equivalent of a kidney, the Malpighian tubules^54^. Thereby, we would predict that thiamethoxam should have the highest toxicity to all three bee species compared with the other neonicotinoids, and our results show that it does.

The current study assists in understanding how neonicotinoid usage patterns may have contributed to changes in worldwide bee populations. All three managed bees can be exposed to and affected by the second generation neonicotinoids, clothianidin and thiamethoxam, while only the bumble bee is exposed to, and affected by, the first generation neonicotinoid, imidacloprid. Although leafcutter bees have the potential for adverse effects from neonicotinoid exposure, this managed bee species is protected from risk by management practices.

## Methods

### Bees

#### i. Worker bumble bees

Commercial nest boxes (*B. impatiens*) or colonies were purchased from Koppert Biological Systems (Scarborough, ON, Canada) in 2015 and 2016. The lids were removed and the nest boxes placed at 26–28 °C degrees, 16:8 photoperiod for an acclimation period of 7 days. We applied a photoperiod to engage the bee clock or circadian rhythm which is known to synchronize bee social behavior and locomotory activity^55^. In addition, photoperiod can improve bee survival under artificial non-social experimental conditions^56, 57^.

#### ii. Summer worker honey bees

Bees were collected from *A. mellifera* colonies maintained in commercial and non-commercial bee yards in southern Alberta, Canada. A frame from a randomly selected colony was removed after brushing off adults and marked with the colony number. This was placed into a mesh sac which was then closed with a zipper and transported back to the laboratory. Frames were then placed in a frame box that was vented and maintained at 28–30 °C, 16:8 photoperiod until emergence The rational for photoperiod is provided in section *i*.

#### iii. Winter worker honey bees

Winter bees that were approximately six months of age were collected by gently touching the winter bee cluster on a frame with the lip of the cage using colonies located in commercial and non-commercial bee yards in southern Alberta, Canada. The bees were collected from March 3 to March 27, 2016 starting with the date the winter wrap was removed. There was no brood production evident during this time period. The cages were taken back to the laboratory and immediately used in acute toxicity studies.

#### iv. Winter worker honey bee anatomy

Any leftover cages from section iii, were used to examine the gross anatomy of winter bees (145 bees) prior to receiving 2:1 honey water with or without neonicotinoid. For each neonicotinoid, a minimum of 10 bees per dose (240 bees) were examined at the end of the experiment to determine if there was an obvious change in tissue regionalization or size to account for changes in susceptibility.

#### v. Leafcutter bees

Overwintered cocoons (*M. rotundata*) that had been collected from different regions in southern Alberta were provided by The Cocoon Testing Centre (Alberta Alfalfa Seed Commission) and from commercial growers. These cocoons were bagged and labeled with a number representing the region within Alberta. Overwintered cocoons were placed in plastic boxes with a metal screen in the lid for ventilation. The boxes were maintained at 28–30 °C, 16:8 photoperiod for 15 days. Then water was added to increase the humidity by placing two soaked pieces of paper towel into a large weigh boat and placing this on top of the cocoons. The boxes were maintained at 28–30 °C, 16:8 photoperiod for an additional 15 days and the paper towels were replaced every 2 days or as required. Peak adult emergence falls within 30 to 45 days. Preliminary experiments indicated no difference in male and female susceptibility to neonicotinoids (data not shown).

### Neonicotinoids

Analytical standard grade Pestanal® acetamiprid, imidacloprid, clothianidin and thiamethoxam were obtained (Sigma-Aldrich, MO, USA). Although some studies suggest that neonicotinoids are soluble in water, precipitation was a problem. To ensure that we were achieving the desired doses, stock solutions were made up in 100% dimethylsulfoxide (DMSO) and stored at −20 °C. Serial dilutions of each chemical were made in 2:1 honey water (honey bees and bumble bees) or 1:1 honey water (leafcutter bees) and the dose range is provided in Table 1. Previous studies have shown that the feeding behavior of worker honey bees is not influenced by 0.1–1% DMSO treatment. We also examined whether the 5% DMSO present in the highest applied dose, would affect bee survival in acute toxicity studies. There was no effect on bee survival compared with bees receiving 2:1 honey water alone for 24 hr, except that early-onset symptoms developed faster. Normally, we would not use 5% DMSO, but one neonicotinoid, acetamiprid, was relatively non-toxic in the preliminary acute toxicity studies, so we included the 5% DMSO:50 µg/µl dose in order to achieve 100% mortality which facilitated statistical analysis.

**Table 1:** Concentrations of four neonicotinoids applied in acute and chronic toxicity bioassays with three bee species.

Neonicotinoid	Control	Dose 1	Dose 2	Dose 3	Dose 4	Dose 5	Dose 6	Dose 7	Dose 8
Acetamiprid	0	11.25 mM	0.56 mM	28.12 µM	1.41 µM	70 nM	3.52 nM	170 pM	8.7 pM
Clothianidin	0	10 mM	0.5 mM	25 µM	1.25 µM	62.5 nM	3.15 nM	156.25 pM	7.81 pM
Imidacloprid	0	9.8 mM	0.49 mM	24.5 µM	1.22 µM	61.25 nM	3.06 nM	153.12 pM	7.65 pM
Thiamethoxam	0	8.55 mM	0.43 mM	21.3 µM	1.06 µM	53.43 nM	2.67 nM	133.59 pM	6.68 pM
all	0	50 µg/µl	2.5 µg/µl	125 ng/µl	6.25 ng/µl	312.5 pg/µl	15.6 pg/µl	0.78 pg/µl	0.039 pg/µl

### Food

In the current study, we eliminated syrup as the food source as beet root,tops or sugar are known to contain residue tolerances of up to 0.3 ppm or 0.3 ng/µl clothianidin or 0.05 ppm or 0.05 ng/µl thiamethoxam. These values fall within the dose ranges where we observed late-onset neurological impairments in all three bee species and we wished to control for this factor in our experiments (https://www3.epa.gov/pesticides/chem_search/cleared_reviews/csr_PC-044309_16-Oct-07_a.pdf; http://publications.gc.ca/collections/collection_2011/sc-hc/H113-29-2011-45-eng.pdf; https://www.canada.ca/en/health-canada/services/consumer-product-safety/pesticides-pest-management/public/consultations/proposed-maximum-residue-limit/2015/clothianidin/document.html). We tested six types of honey to determine which brands produced no mortality or neurological symptoms over14 days. Although most Canadian honeys were acceptable, all but one, facilitated the growth of bacteria and fungi within a few days of the bees feeding on the honey water, Natural Honey Farms (McCormick Canada), Canada No.1 Pasteurized Liquid Honey. Honey water was produced by mixing the honey with an appropriate amount of ultrapure water (EMD Millipore, ON, Canada, Direct-Q3^®^ ultrapure water (Type 1), 18.2 MΩ • cm at 25 °C), to eliminate water as a potential source of contaminating pesticides. Preliminary laboratory trials confirmed that a 2:1 honey water solution was readily consumed by bumble bees and honey bees, while a 1:1 honey water solution was readily consumed by leafcutter bees. In each experiment, the volume of honey consumed per bee was calculated by counting the number of bees in the cage and dividing it by the total volume removed from the feeding tube minus the volume absorbed by the gauze. The average amount consumed per bee was estimated based upon three replicates for each neonicotinoid at all applied doses.

### Acute toxicity bioassays

#### i. Worker bumble bees

Colonies were cooled for 5–8 min at −80 °C, and individual workers were removed using feather-lite forceps as they emerged from the cotton-batting overlaying the brood. Cages consisted of a Fisherbrand™ 9 cm circle of filter paper placed into the bottom of a 1000 ml transparent polypropylene container with insect screening hot glued to a window on the side of the container for ventilation. A bundle of 5–2 inch segments of large straws and a handful of polyester stuffing were then added. Each cage was randomly assigned to a neonicotinoid treatment or untreated control (Table 1). Honey-water (2:1) containing the experimental test solutions were aliquoted to 1 ml Eppendorf tubes, and covered with sterile gauze that was held in place with elastic. The tube was placed into the cage using 25 cm forceps and placed on its side so that it was wedged against the polyester batting. After 24 hr, the tube was removed and the volume consumed recorded. Next, 10 ml of 2:1 honey water was added to a 15 ml centrifuge tube to be consumed ad libitum and covered with sterile gauze held in place with elastic. This tube was passed through a hole in the lid and positioned about 2.5 cm from the polyester batting using elastic wrapped around the tube base. Mortality was recorded at 24, 48, 72, and 96 h, as dictated in the OECD guidelines, but was also extended to include 10 additional days. Early and late-onset neurological impairments are a hallmark for insecticide toxicological studies with pest insects. Neurological impairments were recorded at 24, 48, 72, 96 h, as indicated in the OECD guidelines, but also extended to include 10 additional days. The volume of the dose consumed by the bees was determined and varied with the smallest amount consumed at milimolar concentrations (55 µl/worker) and the highest amount consumed below nanomolar concentrations (120 µl/worker). We used the highest volume consumed for all calculations. Six replicates were performed with each replicate representing a different colony or nest box. Five to nine bees were added to each cage with an average of 30 to 48 bees used in a replicate. The total number of bumble bees used in the bioassay was 1,064.

#### ii. Summer worker honey bees

For each experiment, a mesh sleeve containing one frame was opened and about 50 bees vacuumed up (Insect Vacuum, BioQuip Products, CA, USA) as they approached the zippered opening. The collected bees were then transferred to a 1000 ml transparent polypropylene cage lined with a Fisherbrand™ 9 cm circle of filter paper and a coarse floor vent filter that served as a perch. The cage also had insect screening hot glued to a window on the side of the container for ventilation. Honey water containing the test solutions were placed in 5 ml polypropylene Eppendorf microcentrifuge tubes and covered with sterile gauze held in place with elastic. The tube was then snapped into a hole in the cage lid. The doses delivered are provided in Table 1. After 24 hr, the test solutions were removed and the volume consumed recorded. Post-treatment, 2:1 honey water was provided ad libitum, in a 20 ml scintillation vial and covered with gauze held in place with elastic. This was inserted into a hole in the lid. Mortality was recorded at 24, 48, 72, and 96 h, as dictated in the OECD guidelines, but was also extended to include 10 additional days. As reported for other bee species, the volume of the dose consumed by the bees varied with the smallest amount consumed at milimolar concentrations (39.4 µl/worker) and the highest amount consumed at nanomolar concentrations (66 µl/worker). We used the highest volume consumed for all calculations. Early and late-onset neurological impairments are a hallmark for insecticide toxicological studies with pest insects. Neurological impairments were recorded at 24, 48, 72, 96 h, as indicated in the OECD guidelines, but also extended to include 10 additional days. Six replicates were performed with each replicate representing a frame from a different colony. The vacuuming process did cause cage to cage variation in bee number, but was less stressful than handling the individual bees. To overcome this variation, we applied a volume of honey water in acute toxicity studies to ensure that each bee could consume 66 µl in 24 hr. At the end of each experiment a total bee count was taken for each cage. An average of 34–47 bees were allocated to a cage with an average of 315 to 423 bees used in a replicate. The total number of honey bees used in the bioassay was 8,951.

#### iii. Winter worker honey bees

The same methods were employed as described for summer worker bees (section ii), except winter worker honey bees were directly collected into cages at the colony. Unlike the summer worker honey bees, the volume of the dose consumed by the winter bees did not vary (53 µl/worker)and was used for all calculations. Six replicates were performed with each replicate representing a frame from a different colony. An average of 57–72 bees was allocated to each cage with an average of 513 to 648 bees used in a replicate. The total number of winter bees used in the bioassay was 10,498.

#### iv. Leafcutter bees

For each acute toxicity experiment, 25 newly-emerged bees were transferred to 1000 ml transparent polypropylene cage, lined with a prewashed moisture pad. The cage also had insect screening hot glued to a window on the side of the container for ventilation. A small bundle of 7.5 cm straws wrapped with elastic was placed into the cage and finally one piece of tulle was added as a perch. The honey water (1:1) containing the test solutions was placed in a 15 ml centrifuge tube and covered with gauze held in place with an elastic. The tube was then inserted through a hole in the lid and held in place about 5 cm from the cage bottom using elastic wrapped around the tube base. The doses delivered are provided in Table 1. After 24 hr, the test solutions were removed, the volume consumed recorded and then, replaced with a 15 ml centrifuge tube containing 10 ml of 1:1 honey water to be consumed ad libitum. Mortality was recorded at 24, 48, 72, and 96 h, as dictated in the OECD guidelines, but was also extended to include 10 additional days. Early and late-onset neurological impairments are a hallmark for insecticide toxicological studies with pest insects. Neurological impairments were recorded at 24, 48, 72, 96 h, as indicated in the OECD guidelines, but also extended to include additional assessments for 10 days. Water was added to the sponge at the bottom of the cage as required to maintain a high humidity. The volume of the dose consumed by the bees varied with the smallest amount consumed at milimolar concentrations (16 µl/adult) and the highest amount consumed at nanomolar concentrations (34 µl/adult). We used the highest volume consumed for all calculations. Six replicates were performed with each replicate representing bees collected from different areas in Alberta, Canada. Twenty-five bees were allocated to each cage with 225 bees used in a replicate. The total number of leafcutter bees used in the bioassay was 5,400.

### Chronic toxicity assays

In chronic toxicity studies, we applied the lowest four doses provided in Table 1. The assays were performed using the same procedures described for the acute toxicity assays except that the bees were allowed to consume ad libitum, the test solutions over 14 days. We recorded changes in bee survival and neurological impairment daily for 14 days. We extended the time period to ensure that late-onset neurological dysfunctions would not be missed and also to ensure that recovery could be documented. During the experiments, feeders were changed weekly or as required with fresh honey water containing the test neonicotinoid or no treatment. Consumption rates did not differ between control and neonicotinoid –treated honey water. To avoid photodecomposition, opaque feeder tubes were used together with lightweight curtains. Survival was recorded as dictated in the OECD guidelines for 10 days, but was also extended to include 4 additional days. The total number of leafcutter bees used in the bioassay was 3,000.

### Symptom assessments

Orally-challenged bees were monitored daily for the impact of neonicotinoids using behavioral assessments to identify time-based neurological symptoms (Table 2).

**Table 2:** Time-based neurological symptoms observed in bees after exposure to neonicotinoids.

Onset Time	Behavioral assessment	Neurological symptom
Acute early-onset (minutes to 24 hr)	adults were considered paralyzed when they fell to the floor of the cage and remained immobile after being touched or handled	paralysis
Acute	visual	tremor, excessive grooming, proboscis extension
Acute	presence of salivary droplets or “spitting” bees	salivation
Acute	honey sac contents extruded	vomiting
Acute	movements and bee placement within the cage “straight rows or clusters”	slow to no movement
Acute	the impaired bee was picked up with tweezers and dropped to see if they resumed a normal stance or activities	no righting behavior
Acute and delayed late-onset (3–10 days)	time in motion	hyperactivity
Delayed	bees under the filter paper and do not leave that area for food or as part of daily movements	“lost” orientation
Delayed	wing placement righting behavior (see above) leg placement movement (abnormal gait)	ataxia

### Gross anatomy assessments of winter bees

Each winter bee was placed on a wax dissecting dish, their wings and legs removed. The abdomen and thoracic terga were cut longitudinally near the lateral margins of the bee with cuticle scissors. The bee was pinned ventral side down in the dissecting dish and enough Dulbecco’s Phosphate Buffered Saline (Sigma-Aldrich, Oakville, ON, Canada) added to cover the body. Using scissors, a cut was made across the base and top of the abdomen and thorax. Using pins, the terga were gently moved to one side and pinned down. The median dorsal vessel or heart, tracheae/air sacs, thoracic muscles, fat body, foregut, midgut and hindgut were examined. Each tissue was scored as the same or different (regionalization or no structure; no differentiated cells, tissue size) from what we encountered in summer honey bees or relative to the previous week of winter honey bees. A total of 145 winter bees were dissected as they came into the lab and 240 winter bees were dissected after delivery of the neonicotinoids.

### Statistical analysis

For acute toxicity studies, the LD_50_ values were calculated using the Dr. Fit program^58^ (within R stats program) that uses a modified model and automated fitting procedure to describe dose-response curves with monotonic and multiphasic features. The resulting general model enables interpreting each phase of the dose-response as an independent dose-dependent process. The data was uploaded from excel files and automatically modelled. Each data set was classified as a monophasic or multiphasic dose-response curve based on the goodness of fit statistic. The program calculates LD_50_ values for each peak in the multiphasic curves. To establish whether colony or region was a covariate predicting the response of bees to neonicotinoid exposure, data were square-root transformed to normalize the data prior to analysis with ANCOVA.

For chronic toxicity studies, data were square-root transformed to normalize the data prior to analysis with ANCOVA, which evaluated whether population means for the proportion of bees alive were equal across dose levels, while statistically controlling for the effects of a covariate, nest box or colony. After the analysis was complete, we performed pairwise comparisons of the means using the Bonfferoni test which determined which mean doses differed from untreated or control means.

The datasets generated and analysed during the current study are available from the corresponding author upon reasonable request.
